# Extensive Metastatic Cholangiocarcinoma Associated With IgG4-Related Sclerosing Cholangitis Misdiagnosed as Isolated IgG4-Related Sclerosing Cholangitis: A Case Report and Literature Review

**DOI:** 10.1097/MD.0000000000002052

**Published:** 2015-11-13

**Authors:** Yi-An Zhang, Xi-Zhong Shen, Ji-Min Zhu, Tao-Tao Liu

**Affiliations:** From Department of Gastroenterology, Zhongshan Hospital of Fudan University, Shanghai, China (YAZ, XZS, JMZ, TTL); Shanghai Institute of Liver Diseases, Zhongshan Hospital of Fudan University, Shanghai, China (XZS); and Key Laboratory of Medical Molecular Virology, Shanghai Medical College of Fudan University, Shanghai, China (XZS).

## Abstract

As cholangiographic features of IgG4-related sclerosing cholangitis (IgG4-SC) resemble those of cholangiocarcinoma, it is highly confusing between the 2 conditions on the basis of cholangiographic findings. This study presents a case of extensive metastatic cholangiocarcinoma with IgG4-SC misdiagnosed as isolated IgG4-SC, and reviews recent studies of the 2 diseases.

A 56-year-old man with no family history of malignant tumors or liver diseases presented with recurrent mild abdominal pain and distention for 3 months. Magnetic resonance cholangiopancreatography showed a 3.7 cm nodular lesion with unclear boundary in segment VI of the liver. Serum IgG4 and CA19-9 were slightly elevated. Histopathological examination was consistent with the consensus statement on the pathology of IgG4-SC. IgG4-SC was thus considered. Due to his mild symptoms, glucocorticoid was not given at first. However, 3 months after his first admission, he had more severe abdominal pain and further elevated serum CA19-9. Actually he was found suffering from extensive metastatic cholangiocarcinoma with IgG4-SC by exploratory laparotomy.

The present case serves as a reminder that extensive metastatic cholangiocarcinoma with or without IgG4-SC may be misdiagnosed as an isolated IgG4-SC case if one relies solely on elevated serum and tissue IgG4 levels. We emphasize on the importance of repeated core needle biopsy or exploratory laparoscopy/laparotomy before immunosuppressive drugs are given, and on follow-up of imaging findings and serum CA19-9 once immunosuppressive therapy is started.

## INTRODUCTION

Cholangiocarcinoma, first reported by Durand-Fardel in 1840,^1^ accounts for the second most common primary hepatic tumor worldwide.^2^ It is a difficult-to-diagnose condition that usually presents late, and is associated with a high mortality. Characteristic features of the entity include abdominal pain, biliary strictures, and jaundice.^3^ Although unspecific, serological studies, such as serum bilirubin, liver enzymes (alkaline phosphatase [ALP], γ-glutamyl transpeptidase, and the transaminases), tumor markers (carbohydrate antigen 19-9 [CA19-9] and carcinoembryonic antigen [CEA]), are useful as a diagnostic guide.

IgG4-related sclerosing cholangitis (IgG4-SC) is a new emerging disease which was identified by Hamano et al at 2001.^4^ Given that fact that clinical cholangiographic features of IgG4-SC resemble those of cholangiocarcinoma,^5,6^ it is highly confusing between the 2 conditions in cholangiographic findings. Herein, we describe a case of extensive metastatic cholangiocarcinoma with IgG4-SC that was wrongly diagnosed as isolated IgG4-SC solely on the basis of serum and tissue IgG4 levels, and conduct an overview of the published literature on this issue.

## CASE REPORT

A 56-year-old man with no family history of malignant tumors or liver diseases presented with recurrent mild abdominal pain and distention for 3 months. Duration of pain may last from hours to days, and the pain caused a loss of appetite. He lost 3 kg in body weight in half a month before admission. Physical examination showed mild tenderness in the upper abdomen, without rebound tenderness, guarding, mass, or hepatomegaly. Serum tests revealed that slightly elevated IgG4 (216 mg/dL; normal range, 3–200 mg/dL) and CA19-9 (48.06 U/mL; normal < 37 U/mL). Total bilirubin, conjugated bilirubin, and liver function parameters, including alanine aminotransferase (ALT), aspartate aminotransferase (AST), ALP, and albumin, remained normal (Table 1) by the time of admission.

**TABLE 1:**
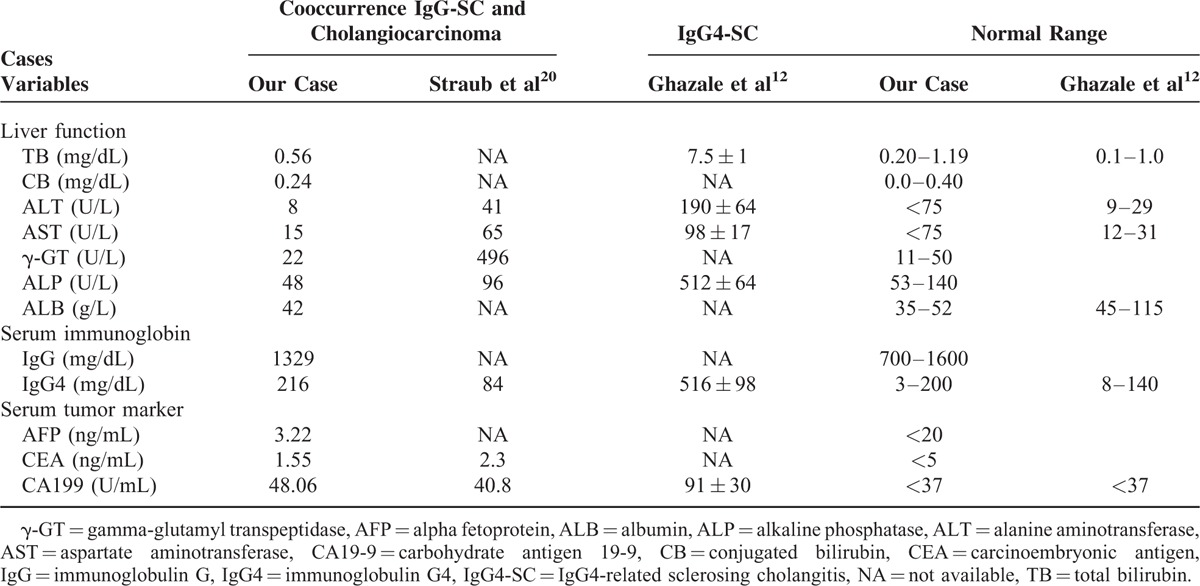
Comparison of Patient's Laboratory Data

**FIGURE 1:**
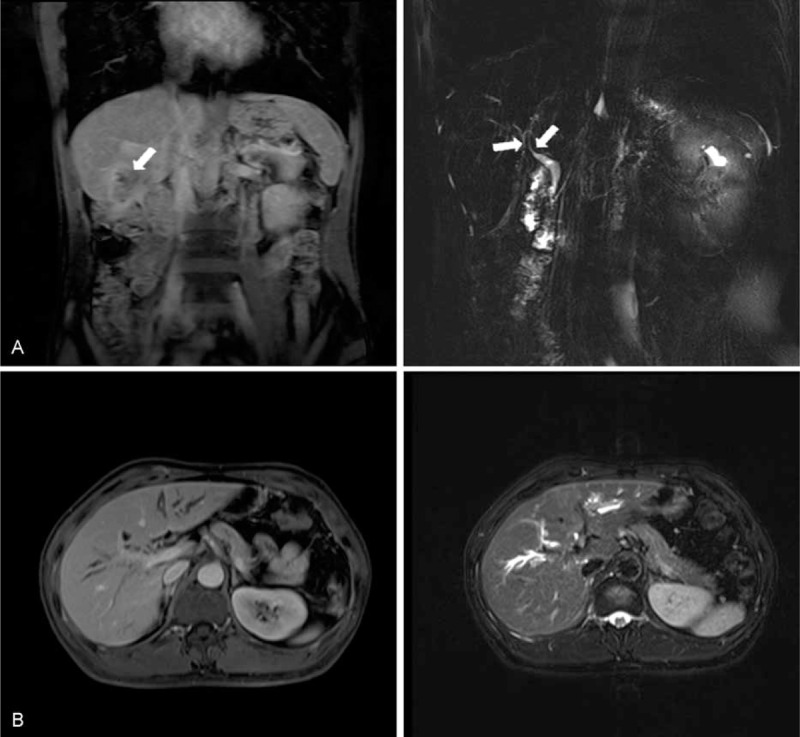
Preoperative magnetic resonance cholangiopancreatography images. (A) Coronal reconstructed image revealed an irregular and abnormal signal intensity lesion (approximately 3.7 cm) in segment VI of liver mimicking malignancy (left), and the obstructed bile duct (right). Arrows show the stricture of the intrahepatic bile ducts caused by the cholangiocarcinoma. (B) Transversal sections on T1-weighted (left) and T2-weighted (right) images showed a slight enhancement of the common bile duct. Dilation and irregularity of biliary tracts were also obvious.

Ultrasonography of the abdomen demonstrated chronic cholecystitis and heterogeneous echogenicity in the liver. Next, magnetic resonance cholangiopancreatography (MRCP) investigation showed a solid nodular lesion at 3.7 cm in size with unclear boundary in segment VI of the liver (Fig. 1A, left). This mass-forming lesion caused obstructive intrahepatic bile duct (Fig. 1A, right). Transversal sections with T1-hypointense (Fig. 1B, left) and T2-hyperintense (Fig. 1B, right) showed a moderate enhancement of the common bile duct. Dilation and irregularity of intrahepatic bile duct were also obvious. Moreover, no enlarged retroperitoneal lymph node was found.

**FIGURE 2:**
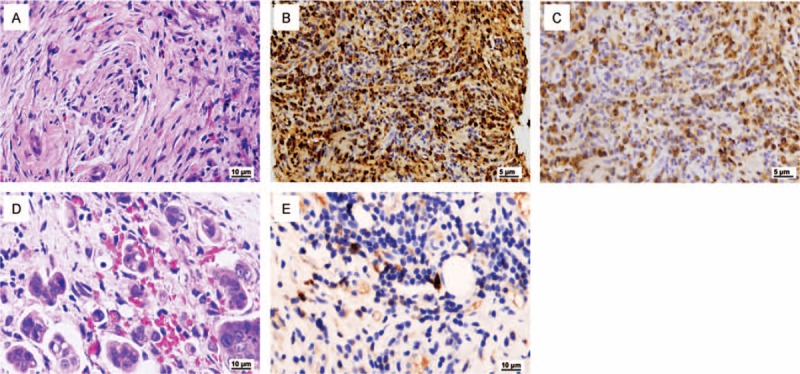
Representative pathological findings. (A) Hematoxylin and eosin (H&E) stain of liver biopsy specimens showed storiform fibrosis, which is characterized by a cartwheel sign with spindle cells radiating from a center and a dense lymphoplasmacytic infiltration (×200). (B) Immunohistochemical staining of liver biopsy specimens for IgG4 (×100). (C) Immunohistochemical staining of liver biopsy specimens for IgG (×100). (D) H&E stain showed gallbladder tissue with metastatic adenocarcinoma cells (×200). (E) Immunohistochemical staining of gallbladder adenocarcinoma tissue for IgG4 staining (×200).

Based on the imaging results, a liver core needle biopsy under ultrasound guide was performed with the aim to exclude malignancy. Histopathological examination revealed conspicuous inflammatory cell infiltration consisting largely of plasmacytes, macrophages, and lymphocytes on a background of fibrous interstices with fibrosis and fibroblast proliferation. Plasmatic infiltrates were particularly apparent, with a portion showing atypia such as polynuclear cells, and represented reactive growth. Proliferative storiform fibrosis was found, which is characterized by a cartwheel sign with spindle cells radiating from a center (Fig. 2A). Immunohistochemistry revealed that the proportion of IgG4/IgG-positive plasma cells was 59.3% (32/54; Fig. 2B and C). These histologic findings were consistent with the consensus statement on the pathology of IgG4-SC,^7^ and therefore, IgG4-SC was highly considered as a diagnosis.

In the view of his slight symptoms, glucocorticoid was not given at first. Considering that liver-occupying lesion and chronic cholecystitis may further lead to abnormal liver function, treatment was initially started with liver protective drug (silymarin, containing about 60% polyphenole silibinin, has a protective effect on hepatocytes and anti-inflammatory effect on bile ducts and gallbladder).^8–10^ However, 3 months after his first MRCP, he had more severe abdominal pain and further elevated serum CA19-9, and was admitted to our hospital again. At this time, the results of MRCP were similar to that of the first one. But enlargement of supraclavicular lymph nodes was suspected by physical examination, which was confirmed by ultrasonography examination (largest 0.7 cm). Due to suspected spread of malignancy accompanied with intraabdominal inflammation and adhesion, laparoscopy was not considered to avoid unnecessary pain and expense. Thus, an exploratory laparotomy in the liver was performed in the surgery department of this hospital.

During the surgery, a tumor at 5 cm in size in the segment VI of the liver was found to invade into the whole layer of the duodenum and transverse colon wall, as well as the serous membrane of the gallbladder wall. Diffuse miliary nodules were observed on abdominal wall, parietal peritoneum, omentum, and diaphragm (Table 2). These nodules under microscopy were featured with typical metastatic adenocarcinoma, while heterocysts were observed in the removed gallbladder that confirmed as poorly differentiated adenocarcinoma (Fig. 2D). Significantly, immunohistochemical staining of IgG4 was also positive for tumor specimens (Fig. 2E). The patient then received palliative treatment and died from multiorgan failure secondary to systemic metastasis 3 months later, and the final diagnosis was extensive metastatic cholangiocarcinoma with IgG4-SC.

**TABLE 2:**
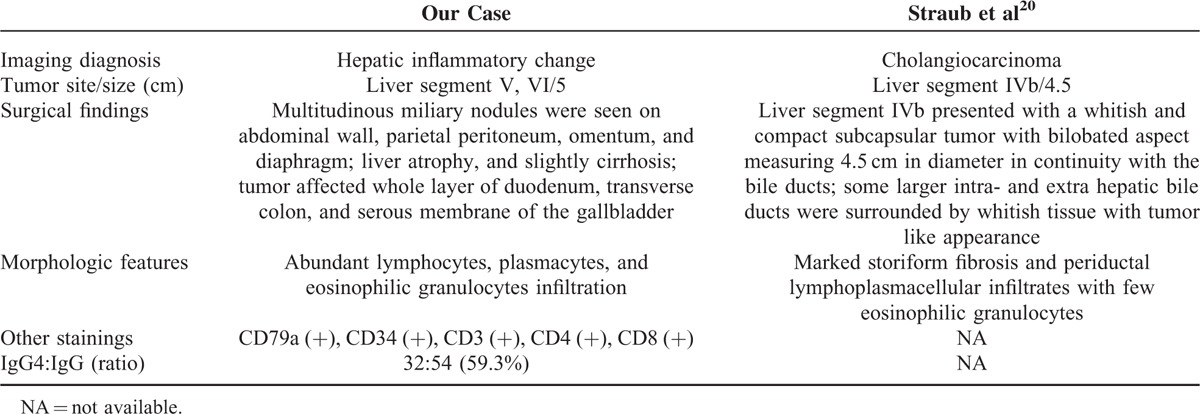
Comparison of Patient's Imaging, Surgical, and Pathological Findings

Institutional approval of what was given by Institutional Ethics Committee of Zhongshan Hospital of Fudan University (Shanghai, China), and informed consent was given by the patient.

## DISCUSSION

IgG4-related disease (IgG4-RD) is a fibroinflammatory, multiorgan condition of unknown etiology with commonly shared features that include elevated serum IgG4 concentrations, tumefactive lesions, storiform fibrosis, as well as dense lymphocyte and IgG4-positive plasmacyte infiltrates.^11^ Clinically, symptoms (including pruritus, weight loss, abdominal distention or pain, and obstructive jaundice), are all nonspecific, and could also be found in both IgG4-SC and cholangiocarcinoma.^12^ It is not easy to distinguish IgG4-SC from primary sclerosing cholangitis (PSC), pancreatic cancer, and cholangiocarcinoma on the basis of cholangiographic findings alone.^13–15^ Radiologic features of IgG4-SC, such as thickening bile duct wall, mimic that of cholangiocarcinoma, and other inflammatory or immune-mediated disorders, such as PSC.^16–18^ For laboratory tests of IgG4-SC, abnormal liver functions, such as elevated bilirubin (65%), ALP (84%), ALT (62%), and AST (32%), could be seen.^12^ However, these abnormal values varied widely among IgG4-SC patients, thus it is hard to find a proper cut-off value to differentiate IgG4-SC from cholangiocarcinoma.

In spite of large similarities in clinical, imaging, and biochemical characteristics, notably, there are still significant differences between IgG4-SC and cholangiocarcinoma (Table 3). Two gastrointestinal symptoms (including steatorrhea and abdominal pain), rarely reported in patients with IgG4-SC,^12^ were presented in this case. The pathophysiology is unclear but seems to reflect a direct gastrointestinal effect in this part of patients and in our case. However, jaundice was absent in our case, which was presented in most IgG4-SC patients. It is possible that IgG4-SC accompanied with autoimmune pancreatitis increases the chance of obstructive jaundice. Further, tumor was found in segment VI of the liver, while the intrahepatic bile ducts were partly dilated in our case. This may partly explain normal total bilirubin and conjugated bilirubin.

**TABLE 3:**
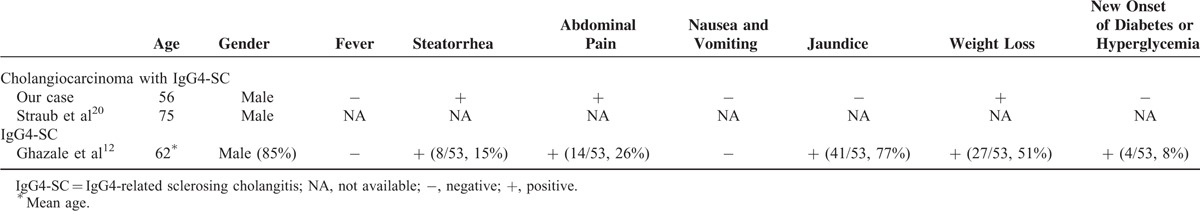
Comparison of Patient's Characteristics

Although molecular mechanisms for elevated serum IgG4 and IgG4-positive plasma cells remains largely unknown, serum IgG4 level could be considered as a noninvasive and proven test for the diagnosis of IgG4-SC.^19^ In our study, serum IgG4 level of this patient was slightly rising (216 mg/dL); while Straub et al^20^ reported that serum IgG4 level (84 mg/dL) is within the normal range in the co-occurrence of cholangiocarcinoma with IgG4-SC. By comparison, a cross-sectional study of 53 IgG4-SC patients found that mean serum IgG4 level is 516 ± 98 mg/dL (mean ± SE), and the level over 280 mg/dL accounts for up to 50%.^12^ It seems that serum IgG4 level in cholangiocarcinoma is not as high as that in IgG4-SC. Ohara et al^21^ conducted a cohort study to establish a cut-off value for IgG4 level with the expectation of distinguishing IgG4-SC from other non-IgG4-related hepatopancreatobiliary diseases. The cut-off level of serum IgG4 was set at 135 mg/dL to differentiate all the 344 IgG4-SC patients from pancreatic cancer, PSC, and cholangiocarcinoma controls. However, it is noteworthy that the specificity of the value is low for distinguishing IgG4-SC from cholangiocarcinoma, especially those with cholangiocarcinoma and PSC. Moreover, Oseini et al^22^ suggested that serological IgG4 concentrations above 4 times the upper limit of the normal range is more specific for IgG4-SC.

**FIGURE 3:**
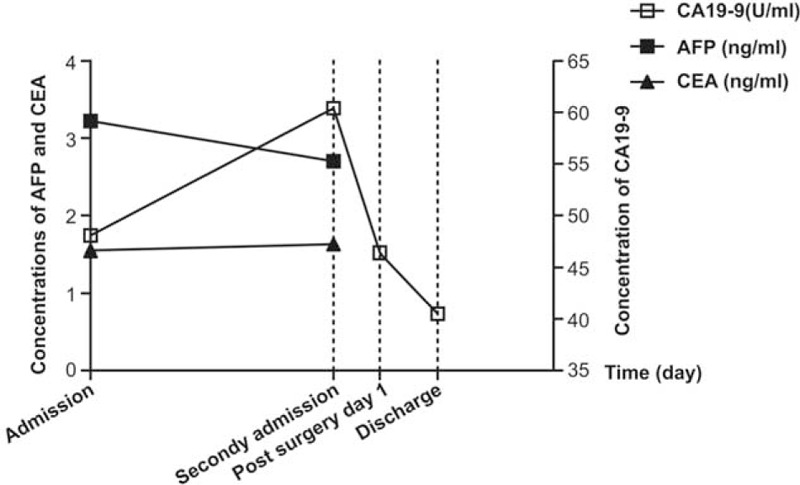
Dynamic changes of serum tumor marker: serum carbohydrate antigen 19-9 (CA19-9), alpha-fetoprotein (AFP), and carcinoembryonic antigen (CEA). Serum CA19-9 level showed a sustained elevation before surgery and then a rapid drop after surgery. Serum AFP and CEA remained normal between the interval of the first and the second admission.

Serum CA19-9 has long been used as a prognostic indicator for various cancer patients (including pancreatitis, colon cancer, and bile duct diseases) during follow-up evaluations after surgery, radiotherapy, and/or chemotherapy.^23–25^ It was also elevated both in IgG4-SC and in cholangiocarcinoma (Table 1).^26^ Therefore, efforts were made to set up a proper cut-off value to differentiate cholangiocarcinoma from other diseases. However, according to a previous study conducted by Li et al,^12^ average CA19-9 level was 91 ± 30 U/mL in cholangiocarcinoma, while over 37 U/mL accounting for 48% and over 100 U/mL accounting for 18%. Further, a study reported by Levy et al^27^ found that the sensitivity and specificity are 78.6% and 98.5%, respectively, at the cut-off value of 129 U/mL for cholangiocarcinoma, although the positive predictive value is only 56.6%. In this study, serum CA19-9 level for this 56-year-old male was 148.06 U/mL at admission. A continuous elevation was noted after he was diagnosed with IgG4-SC; however, a rapid reduction was then observed 1 day after surgery (Fig. 3). Additionally, AFP and CEA levels remained normal before surgery. We infer that sustained elevation of serum CA19-9 may imply the occurrence of tumor in hepatobiliary system of IgG4-RD, and subsequent decline could be due to gallbladder resection. Since the existence of the difficulty to define a proper cut-off value and of the poor diagnostic power of serum CA19-9, the dynamic monitoring of serum CA19-9 may be, in our opinion, used to follow-up patients on immunosuppressive therapy.

Integrated positron emission tomography and computed tomography (PET/CT) with ^18^F-fluorodeoxyglucose (^18^F-FDG) has been widely used in the diagnosis, staging, and relapse monitoring of malignancies. Recently, a few case reports have suggested the value of ^18^F-FDG PET/CT in IgG4-RD diagnosis.^28–30^ Zhang et al^31^ conducted a prospective research in 35 patients underwent ^18^F-FDG PET/CT who were diagnosed with IgG4-RD. The results indicated that ^18^F-FDG uptake lesion in bile duct and liver, without signs of infection, is a moderate indicator for IgG4-SC, and that ^18^F-FDG PET/CT may serve as a powerful tool by assessing disease distribution, thus helping to guide the biopsy site. Further, Ozaki et al^32^ indicated that ^18^F-FDG uptake by the hilar lymph node is significantly more frequent in autoimmune pancreatitis (a major constituent of IgG4-RD) than in pancreatic cancer. Based on the experience in the management of this case, we feel that ^18^F-FDG PET/CT is likely to be one potential key modality in the assessment of the overall condition of patients with IgG-SC. However, ^18^F-FDG PET/CT does not appear to have a well-established role in the diagnosis of patients with IgG4-SC in this case. Future prospective studies are required to define the cost-effectiveness and clinical impact in the management of patients with IgG4-SC.

**FIGURE 4:**
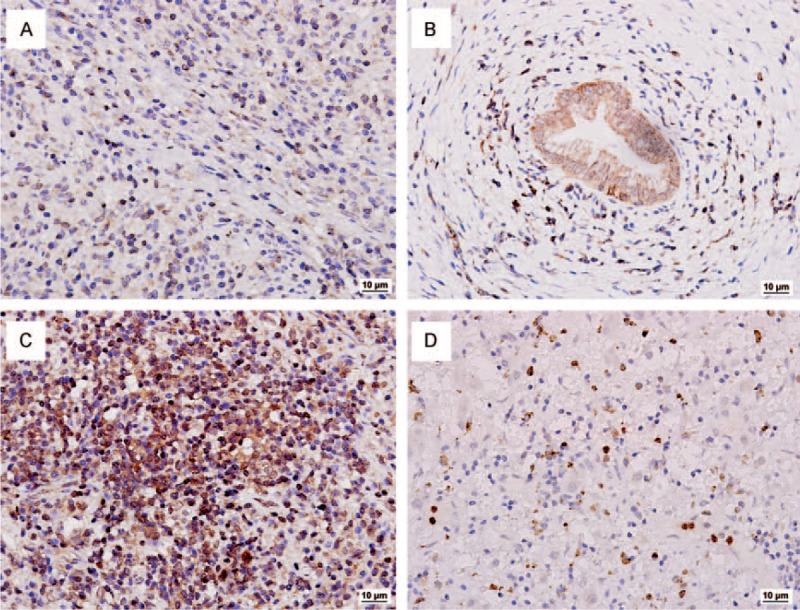
Representative findings of immunohistochemical stainings. (A) Interleukin-4-positive cells scattered in the germinal centers and the Disse space without preference. (B) Interferon-γ-positive cells mainly gathered around the bile ducts. (C) Nuclear expressions of Foxp3-positive cells are scattered in the germinal centers of lymphfollicle of the specimens. (D) Interleukin-17-positive cells scattered in the germinal centers and the Disse space without preference. Original magnification ×200.

It is well known that inflammation contributes to the pathogenesis of cholangiocarcinoma.^33^ A variety of cytokines and growth factors involved in proliferation, apoptosis, senescence, and cell-cycle regulation are required for the development of cholangiocarcinoma genesis. It is well-known that PSC, another autoimmune biliary disease, has been regarded as one of the most common predisposing condition for cholangiocarcinoma with 0.5% to 1.5% of PSC patients developing into cholangiocarcinoma per year.^34^ As a result, the chronic inflammation microenvironment of IgG4-SC may also be involved in the pathogenesis of cholangiocarcinoma. In this case, immunohistochemical staining of interleukin-4 (IL-4, a Th2 type cytokine), IL-17, interferon-γ (INF-γ, a Th1 type cytokine), and Foxp3 (the marker of Tregs) was performed. IL-4^+^ cells scattered in the germinal centers and the Disse space without preference (Fig. 4A), while INF-γ^+^ cells mainly gathered around the bile ducts (Fig. 4B). Generally, IgG4-RD is Th-2 type cytokines dominant,^11^ in other words, the amount of IL-4^+^ cells surpasses that of INF-γ^+^ cells. Despite no expression of INF-γ^+^ cells was reported in previous study of isolated IgG4-SC, a moderate expression of INF-γ^+^ cells was, however, detected in bile ducts of this case, which may be due to the co-occurrence of cholangiocarcinoma and IgG4-SC. Further, markedly elevation of Foxp3^+^ lymphocytes, a specific cell surface marker of Treg,^5^ was scattered in the germinal centers of lymph follicle (Fig. 4C). It is commonly known that the function of Tregs is impaired in classic autoimmune diseases.^35^ In the present study, the activation of Tregs was considered possibly to be a secondary response that prohibits the overreaction of Th2-dominated autoimmunity of IgG4-SC.^36^ Also, the expression of intratumor IL-17^+^ cells (a novel IL-17-producing CD4^+^ T helper cell subset) has not been reported in IgG4-SC previously, but was detected in the germinal centers and the Disse space without preference in this patient (Fig. 4D).^37^ However, the exact immunological mechanism responsible for the changed cytokine profile is not clear, and future studies may warrant defining their functional roles in the pathogenesis of cholangiocarcinoma and/or IgG4-SC.

Only a few studies have reported the cooccurrence of IgG-RD and malignancies within the same organ, such as the cooccurrence of IgG4-SC and cholangiocarcinoma.^20,38,39^ At present, a great deal of effort has been focused in differentiating IgG4-SC from cholangiocarcinoma in order to avoid arbitrary surgical resections. However, misdiagnosis of cholangiocarcinoma as IgG4-SC, on the other hand, resulted in delaying in optimal surgical time, and consequently, the use of steroid drugs in these patients will lead to tumor dissemination. Therefore, what would be the proper management algorithm in this scenario? Once IgG4-SC is diagnosed and the immunosuppressive drug is given, we recommend follow-up of serum IgG4 and CA19-9, as well as the imaging examination such as ultrasound, CT or MRI scanning. Cholangiocarcinoma should be reconsidered if there is no sign of reduction in tumor size, decreased serum IgG4 levels or continuous elevation of serum CA19-9 level after the treatment. At that time, repeated core needle biopsy should be considered. Laparoscopy is also an option to consider when a contraindication does not exist. Further, although not the first choice, ^18^F-FDG PET/CT could be served as an approach to follow-up the therapeutic response of IgG4-SC under the consent of patients.

Curative surgery is the best way to obtain good survival for resectable cholangiocarcinoma. However, chemotherapy was the optimal therapy for this extensive metastatic patient. Combination chemotherapy with gemcitabine and cisplatin has become the practical therapies for cholangiocarcinoma.^40^ Two randomized clinical trials^41^ reported that combination use of the 2 agents had a better survival rate compared with patients using these 2 drugs separately. However, responses of these chemotherapies are limited and the 5-year survival remains low.^42^ Targeted therapy using Lapatinib, a dual epidermal growth factor receptor (EGFR) and human epidermal growth factor receptor type 2 (HER2) kinase inhibitor, was significantly effective in inhibiting of the growth of human cholangiocarcinoma cell lines.^43^ A phase I study is still underway, investigating the role of Dovitinib, small molecule fibroblast growth factor receptor (FGFR) inhibitor, in combination with gemcitabine and capecitabine in patients with biliary cancers.^44^ Additionally, a phase I trial of everolimus, a mammalian target of rapamycin (mTOR) inhibitor, in combination with gemcitabine and cisplatin, points to a promising future for cholangiocarcinoma patients who are refractory to conventional therapies.^45^

## CONCLUSION

In conclusion, we surmised that IgG4-SC and cholangiocarcinoma are not 2 separate entities; investigating whether they share common molecular abnormalities may lead to a better understanding of their relationship. At present, it is hard to make it clear whether these 2 diseases have a causal or just a coincidental association.^5,13^ Thus, although low incidence of the cooccurrence of IgG4-SC and cholangiocarcinoma exits, we highly recommend repeated core needle biopsy or even laparoscopy/laparotomy before immunosuppressive drugs are given in addition to the laboratory test of serum and tissue IgG4 levels. Follow-up of the serum CA19-9 and imaging evidences should continue once immunosuppressive therapy starts. ^18^F-FDG PET/CT should also take into consideration when imaging examinations (such as CT or MRI scanning) fail to help.

## References

[1] Olnes MJ Erlich R A review and update on cholangiocarcinoma. *Oncology* 2004; 66:167–179. 15218306 10.1159/000077991

[2] Khan SA Thomas HC Davidson BR Cholangiocarcinoma. *Lancet* 2005; 366:1303–1314. 16214602 10.1016/S0140-6736(05)67530-7

[3] Jarnagin WR Shoup M Surgical management of cholangiocarcinoma. *Semin Liver Dis* 2004; 24:189–199. 15192791 10.1055/s-2004-828895

[4] Hamano H Kawa S Horiuchi A High serum IgG4 concentrations in patients with sclerosing pancreatitis. *N Engl J Med* 2001; 344:732–738. 11236777 10.1056/NEJM200103083441005

[5] Nakazawa T Ohara H Sano H Cholangiography can discriminate sclerosing cholangitis with autoimmune pancreatitis from primary sclerosing cholangitis. *Gastrointest Endosc* 2004; 60:937–944. 15605009 10.1016/s0016-5107(04)02229-1

[6] Ohara H Okazaki K Tsubouchi H Clinical diagnostic criteria of IgG4-related sclerosing cholangitis 2012. *J Hepatobiliary Pancreat Sci* 2012; 19:536–542. 22717980 10.1007/s00534-012-0521-y

[7] Deshpande V Zen Y Chan JK Consensus statement on the pathology of IgG4-related disease. *Mod Pathol* 2012; 25:1181–1192. 22596100 10.1038/modpathol.2012.72

[8] Vargas-Mendoza N Madrigal-Santillan E Morales-Gonzalez A Hepatoprotective effect of silymarin. *World J Hepatol* 2014; 6:144–149. 24672644 10.4254/wjh.v6.i3.144 PMC3959115

[9] Boigk G Stroedter L Herbst H Silymarin retards collagen accumulation in early and advanced biliary fibrosis secondary to complete bile duct obliteration in rats. *Hepatology* 1997; 26:643–649. 9303494 10.1002/hep.510260316

[10] Hagymasi K Kocsis I Lugasi A Extrahepatic biliary obstruction: can silymarin protect liver function? *Phytother Res* 2002; 16 Suppl. 1:S78–S80. 11933147 10.1002/ptr.786

[11] Stone JH Zen Y Deshpande V IgG4-related disease. *N Engl J Med* 2012; 366:539–551. 22316447 10.1056/NEJMra1104650

[12] Ghazale A Chari ST Zhang L Immunoglobulin G4-associated cholangitis: clinical profile and response to therapy. *Gastroenterology* 2008; 134:706–715. 18222442 10.1053/j.gastro.2007.12.009

[13] Kalaitzakis E Levy M Kamisawa T Endoscopic retrograde cholangiography does not reliably distinguish IgG4-associated cholangitis from primary sclerosing cholangitis or cholangiocarcinoma. *Clin Gastroenterol Hepatol* 2011; 9:800.e2–803.e2. 21699807 10.1016/j.cgh.2011.05.019 PMC3246637

[14] Nakazawa T Naitoh I Hayashi K Diagnostic criteria for IgG4-related sclerosing cholangitis based on cholangiographic classification. *Journal of gastroenterology* 2012; 47:79–87. 21947649 10.1007/s00535-011-0465-z

[15] Nishino T Oyama H Hashimoto E Clinicopathological differentiation between sclerosing cholangitis with autoimmune pancreatitis and primary sclerosing cholangitis. *J Gastroenterol* 2007; 42:550–559. 17653651 10.1007/s00535-007-2038-8

[16] Srivali N Ratanapo S Ungprasert P Significance of lymphadenopathy in IgG4-related sclerosing disease and sarcoidosis. *Chest* 2013; 143:1191–1192. 23546510 10.1378/chest.12-2954

[17] Okazaki K Uchida K Ikeura T Current concept and diagnosis of IgG4-related disease in the hepato-bilio-pancreatic system. *J Gastroenterol* 2013; 48:303–314. 23417598 10.1007/s00535-012-0744-3 PMC3698437

[18] Bosco JJ Suan D Varikatt W Extra-pancreatic manifestations of IgG4-related systemic disease: a single-centre experience of treatment with combined immunosuppression. *Intern Med J* 2013; 43:417–423. 23013529 10.1111/j.1445-5994.2012.02964.x

[19] Koike T IgG4-related disease: why high IgG4 and fibrosis? *Arthritis Res Ther* 2013; 15:103. 23351335 10.1186/ar4122 PMC3672717

[20] Straub BK Esposito I Gotthardt D IgG4-associated cholangitis with cholangiocarcinoma. *Virchows Arch* 2011; 458:761–765. 21484428 10.1007/s00428-011-1073-2

[21] Ohara H Nakazawa T Kawa S Establishment of a serum IgG4 cut-off value for the differential diagnosis of IgG4-related sclerosing cholangitis—a Japanese cohort. *J Gastroenterol Hepatol* 2013; 28:1247–1251. 23621484 10.1111/jgh.12248

[22] Oseini AM Chaiteerakij R Shire AM Utility of serum immunoglobulin G4 in distinguishing immunoglobulin G4-associated cholangitis from cholangiocarcinoma. *Hepatology* 2011; 54:940–948. 21674559 10.1002/hep.24487 PMC3253343

[23] Venkatesh PG Navaneethan U Shen B Increased serum levels of carbohydrate antigen 19-9 and outcomes in primary sclerosing cholangitis patients without cholangiocarcinoma. *Dig Dis Sci* 2013; 58:850–857. 23007734 10.1007/s10620-012-2401-3

[24] Hess V Glimelius B Grawe P CA 19-9 tumour-marker response to chemotherapy in patients with advanced pancreatic cancer enrolled in a randomised controlled trial. *Lancet Oncol* 2008; 9:132–138. 18249033 10.1016/S1470-2045(08)70001-9

[25] Bauer TM El-Rayes BF Li X Carbohydrate antigen 19-9 is a prognostic and predictive biomarker in patients with advanced pancreatic cancer who receive gemcitabine-containing chemotherapy: a pooled analysis of 6 prospective trials. *Cancer* 2013; 119:285–292. 22786786 10.1002/cncr.27734 PMC4261189

[26] Liu SL Song ZF Hu QG Serum carbohydrate antigen (CA) 19-9 as a prognostic factor in cholangiocarcinoma: a meta-analysis. *Front Med China* 2010; 4:457–462. 21191748 10.1007/s11684-010-0240-1

[27] Levy C Lymp J Angulo P The value of serum CA 19-9 in predicting cholangiocarcinomas in patients with primary sclerosing cholangitis. *Dig Dis Sci* 2005; 50:1734–1740. 16133981 10.1007/s10620-005-2927-8

[28] Kitada M Matuda Y Hayashi S IgG4-related lung disease showing high standardized uptake values on FDG-PET: report of two cases. *J Cardiothorac Surg* 2013; 8:160. 23800259 10.1186/1749-8090-8-160 PMC3717047

[29] Taniguchi Y Ogata K Inoue K Clinical implication of FDG-PET/CT in monitoring disease activity in IgG4-related disease. *Rheumatology (Oxford)* 2013; 52:1508. 23674816 10.1093/rheumatology/ket182

[30] Nakatani K Nakamoto Y Togashi K Utility of FDG PET/CT in IgG4-related systemic disease. *Clin Radiol* 2012; 67:297–305. 22119099 10.1016/j.crad.2011.10.011

[31] Zhang J Chen H Ma Y Characterizing IgG4-related disease with (1)(8)F-FDG PET/CT: a prospective cohort study. *Eur J Nucl Med Mol Imaging* 2014; 41:1624–1634. 24764034 10.1007/s00259-014-2729-3 PMC4089015

[32] Ozaki Y Oguchi K Hamano H Differentiation of autoimmune pancreatitis from suspected pancreatic cancer by fluorine-18 fluorodeoxyglucose positron emission tomography. *J Gastroenterol* 2008; 43:144–151. 18306988 10.1007/s00535-007-2132-y

[33] Schottenfeld D Beebe-Dimmer J Chronic inflammation: a common and important factor in the pathogenesis of neoplasia. *CA Cancer J Clin* 2006; 56:69–83. 16514135 10.3322/canjclin.56.2.69

[34] Aithal GP Breslin NP Gumustop B High serum IgG4 concentrations in patients with sclerosing pancreatitis. *N Engl J Med* 2001; 345:147–148. 11450670 10.1056/NEJM200107123450215

[35] Sakaguchi S Ono M Setoguchi R Foxp3 + CD25 + CD4 + natural regulatory T cells in dominant self-tolerance and autoimmune disease. *Immunol Rev* 2006; 212:8–27. 16903903 10.1111/j.0105-2896.2006.00427.x

[36] Belkaid Y Rouse BT Natural regulatory T cells in infectious disease. *Nat Immunol* 2005; 6:353–360. 15785761 10.1038/ni1181

[37] Gu FM Gao Q Shi GM Intratumoral IL-17(+) cells and neutrophils show strong prognostic significance in intrahepatic cholangiocarcinoma. *Ann Surg Oncol* 2012; 19:2506–2514. 22411204 10.1245/s10434-012-2268-8

[38] Harada K Shimoda S Kimura Y Significance of immunoglobulin G4 (IgG4)-positive cells in extrahepatic cholangiocarcinoma: molecular mechanism of IgG4 reaction in cancer tissue. *Hepatology* 2012; 56:157–164. 22290731 10.1002/hep.25627

[39] Kimura Y Harada K Nakanuma Y Pathologic significance of immunoglobulin G4-positive plasma cells in extrahepatic cholangiocarcinoma. *Hum Pathol* 2012; 43:2149–2156. 22647350 10.1016/j.humpath.2012.03.001

[40] Woo SM Lee WJ Kim JH Gemcitabine plus cisplatin versus capecitabine plus cisplatin as first-line chemotherapy for advanced biliary tract cancer: a retrospective cohort study. *Chemotherapy* 2013; 59:232–238. 24356333 10.1159/000354539

[41] Valle JW Furuse J Jitlal M Cisplatin and gemcitabine for advanced biliary tract cancer: a meta-analysis of two randomised trials. *Ann Oncol* 2014; 25:391–398. 24351397 10.1093/annonc/mdt540

[42] Weigt J Malfertheiner P Cisplatin plus gemcitabine versus gemcitabine for biliary tract cancer. *Expert Rev Gastroenterol Hepatol* 2010; 4:395–397. 20678012 10.1586/egh.10.45

[43] Andersen JB Spee B Blechacz BR Genomic and genetic characterization of cholangiocarcinoma identifies therapeutic targets for tyrosine kinase inhibitors. *Gastroenterology* 2012; 142:1021.e15–1031.e15. 22178589 10.1053/j.gastro.2011.12.005 PMC3413201

[44] Escudier B Grunwald V Ravaud A Phase II results of Dovitinib (TKI258) in patients with metastatic renal cell cancer. *Clin Cancer Res* 2014; 20:3012–3022. 24691021 10.1158/1078-0432.CCR-13-3006

[45] Borad MJ Champion MD Egan JB Integrated genomic characterization reveals novel, therapeutically relevant drug targets in FGFR and EGFR pathways in sporadic intrahepatic cholangiocarcinoma. *PLoS Genet* 2014; 10:e1004135. 24550739 10.1371/journal.pgen.1004135 PMC3923676

